# Physiological Performance of Rabbits Administered Buffalo Milk Yogurts Enriched with Whey Protein Concentrate, Calcium Caseinate or *Spirulina platensis*

**DOI:** 10.3390/foods10102493

**Published:** 2021-10-18

**Authors:** Atallah A. Atallah, Ali Osman, Mahmoud Sitohy, Dalia G. Gemiel, Osams H. El-Garhy, Islam H. El Azab, Nadia. H. Fahim, Abdelmoniem M. Abdelmoniem, Amir E. Mehana, Tharwat A. Imbabi

**Affiliations:** 1 Department of Dairy Science, Faculty of Agriculture, Benha University, Moshtohor 13736, Egypt; dalia.gamil@fagr.bu.edu.eg; 2 Biochemistry Department, Faculty of Agriculture, Zagazig University, Zagazig 44511, Egypt; mzsitohy@hotmail.com; 3 Animal Production Departments, Faculty of Agriculture, Benha University, Moshtohor 13736, Egypt; osama.mohamed@fagr.bu.edu.eg (O.H.E.-G.); tharwat.mohamed@fagr.bu.edu.eg (T.A.I.); 4 Food Science & Nutrition Department, College of Science, Taif University, P.O. Box 11099, Taif 21944, Saudi Arabia; i.helmy@tu.edu.sa; 5 Animal Production Department, Faculty of Agriculture, Cairo University, Giza 12613, Egypt; nadiaamn@agr.cu.edu.eg; 6 Poultry Production Department, Faculty of Agriculture, Ain Shams University, Cairo 11566, Egypt; abdelmoniem_hanafy@agr.asu.edu.eg; 7 Department of Zoology, Faculty of Science, Suez Canal University, Ismailia 41611, Egypt; amirelsayed@science.suez.edu.eg

**Keywords:** yogurt, whey protein concentrate, Ca-caseinate, *Spirulina platensis*, rabbit, antioxidant, growth performance, haemato-biochemical changes, histological examination

## Abstract

The present study examines the impacts of supplementing yogurt with 1% whey protein concentrate (WPC), Ca-caseinate (Ca-CN) and Spirulina platensis on the physiological performance of V-line rabbits receiving diets containing yogurt (at a dose of 5 g/kg body weight/day) and the different meat quality aspects. The results show that fat content was highest (*p* < 0.05) in yogurt fortified with Spirulina powder, but protein (%) was highest in yogurt enriched with WPC. Yogurt containing Spirulina powder showed a significant (*p* < 0.05) increase in total antioxidant activity. The final live body weight for G1 was higher than the other groups. However, additives affected the saddle, hind legs, liver and neck percentages significantly (*p* < 0.05). There were not significant differences for all groups in the forelegs, lung and heart percentages. LDL-cholesterol, total protein, globulin, albumin, creatinine and immunoglobulin M values were lowest (*p* < 0.05) in the WPC group. Significant improvements appeared in the small intestinal wall, microbiology, growth performance, serum biochemistry, organ histology and meat quality of the group receiving enriched yogurt. Yogurts enriched with WPC, Ca-CN and *Spirulina platensis* can be used as functional foods.

## 1. Introduction

Fermented milk has attracted increasing attention from health-conscious consumers because of its nutritional values and health-promoting properties [1]. In addition, so has lactic acid bacteria synthesize inhibitory substances such as organic acid, H_2_O_2_ and bacteriocins [2], which can inhibit the action of various pathogenic organisms. Fermented milk products effectively treat diarrhea, especially in infants, and lactose intolerance [2]. Whey protein and skim milk powders are widely used to enrich yogurt. However, the new technology used for separation can produce a wide variety of milk ingredients, such as caseinate (Na or Ca-caseinate) and whey protein concentrates (WPC). They have different properties and can be used separately or blended to replace skim milk powder in yogurt manufacture. Previous studies documented the beneficial impacts of caseinate and WPC used separately in manufacturing nonfat and low-fat yogurts [3]. Dairy products are the main dietary product for animal nutritious proteins that are appropriate sources for sufficient and balanced quantities of amino acids for human tissues [4]. 

Spirulina is a unicellular cyanobacterium within the Oscillatoraceae algae family, commonly used as a food additive rich in proteins, carotenoids, vitamins and minerals. Previous studies reported the beneficial effects of Spirulina against oxidative stress, hyperglycemia, hypercholesterolemia and arterial hypertension [5]. Spirulina is primarily used as a human food supplement based on its potential antiallergenic, antiviral, antioxidant, hepatoprotective and immunomodulatory properties [5,6,7]. Moreover, it is recommended for animal feeds, being rich in polyunsaturated fatty acids, proteins, vitamin B-complex, vitamin C, phycocyanin, minerals and polyphenols [5]. Spirulina powder is gaining acceptance as an excellent prospective functional feed additive. It is qualified as a superfood that impacts growth, antioxidant mechanism, health and life quality. Thus, it is also imperative for cell regeneration and growth. Spirulina powders are edible algae [8,9] it is a highly nutritious feed resource for several important animal types. It improves animal welfare, physiological and health responses and potentially enhances farm animal fertility and reproductive performance, such as in rabbits. Hence, the beneficial aspect of different values of Spirulina fortification on productive performance, physiological status and health response of various farm animals were previously studied [10].

It was also observed that the growth performance of adult New Zealand White (NZW) rabbit bucks that were fed a Spirulina (700 mg) diet significantly improved compared with the control group. Furthermore, Spirulina powder was reported to enhance animal growth performance by improving the immune system [11,12,13]. Nevertheless, many long-term animal aspects indicated that casein might affect the obesity status in mice fed on a high-fat diet [14] and gut microbes in rats fed on a regular fat diet [15], indicating a crucial aspect of the protein sources. Recently, gut microorganisms have been indicated as playing a critical role in human health by affecting energy homeostasis, the immune system or physiology [16,17]. In addition, diet intake can determine the metabolic outputs and diversity of the microbes’ community [18]. Taken together, dietary protein-associated variations in gut microorganisms could be causally linked with host metabolism.

The meat produced by goats, cattle, buffalo, poultry and sheep is insufficient to meet the increasing demand for animal protein. Therefore, it is urgent to explore alternative animal protein sources to minimize the shortage of protein resources. Rabbit, an important micro-livestock, may be considered a promising and potential alternative source of protein in this regard. Meat quality of rabbits improved when rabbits received Spirulina powder. Meineri et al. (2009) and Peiretti and Meineri (2011) identified Spirulina as a modern factor of increasing *n*-6/*n*-3 polyunsaturated fatty acid (PUFA) and *γ*-linolenic acid (GLA) levels in rabbit muscle lipids. Improving rabbit meat’s oxidative stability by Spirulina supplementation may contribute to consumer-preferable meat appearance and color [19]. Furthermore, rabbit health has been reported to improve with dietary Spirulina, as indicated by higher oxyhemoglobin contents than the control [20]. 

The present research study aims to produce new types of yogurt by supplementing buffalo milk with different sources of WPC, Ca-CN and Spirulina powders as growth promoters. The potential influence of these supplementations was traced by studying growth performance, serum biochemical properties, histological examination and meat composition of weaned V-line rabbits.

## 2. Materials and Methods

### 2.1. Materials

The herd of the Faculty of Agriculture of Benha University in Egypt provided the fresh buffalo milk used in this study. Christens Hansen’s Lab., Copenhagen, Denmark, offered the culture strains of *Streptococcus* (Str.) *thermophilus* and *Lactobacillus* (Lb.) *delbrueckii* subsp. *bulgaricus* (1:1). WPC (A235) was obtained from Lacma Company, Poland. Rennet casein powder (Ca-CN) was obtained from Tecnogene Company, Egypt. *Spirulina platensis* (food grade Spirulina) powder was provided by the Aquaculture Research Center (ARC) at the Arab Academy for Science, Technology and Maritime Transport (Alexandria, Egypt). The Spirulina was packaged under vacuum and stored under dark conditions.

The current study was carried out in the Rabbit Research Laboratory of the Faculty of Agriculture, Benha University, Egypt. The local Experimental Animal Care Committee the ethics approved the design and manipulating procedures of the study. Handling animals followed the husbandry guidelines of Benha University standard operating procedures.

### 2.2. Methods

#### 2.2.1. Manufacture of Yogurt

Fresh buffalo milk (BM) was standardized to 1.2% milkfat and divided into four different portions that were studied: G1 (BM with 1% WPC), G2 (BM with 1% Ca-CN), G3 (BM with 1% Spirulina) and Cl+ (BM without any supplements was considered as a positive control). All milk portions were heated to 85 °C/15 min, immediately cooled to 42 °C and combined with 3% starter cultures. The milk portions were transmitted into plastic cups (500 mL) and incubated at 42 °C until the pH reached 4.6 to 4.7 and kept at 5 ± 1 °C. The developed yogurts were analyzed for physicochemical and microbiological characteristics. All experiments were repeated three times successively, and all analyses were performed in duplicate.

#### 2.2.2. Animals and Experimental Design

A total number of 50 V-line four-week-old weaned male rabbits, with similar average body weight (510 ± 10 g) were randomly divided into five groups (*n* = 10) and housed in twenty-five replica cages (45 × 55 × 30 cm), each holding two animals. Five cages were randomly assigned to one of five animal groups. The first four groups received the four versions of yogurt (G1–G3) orally at a dose of 5 g/kg body weight/day. The fourth group receiving the non-supplemented yogurt was considered the positive control (Cl+), while the fifth group receiving only the basal diet was considered the negative control without yogurt (Cl−). The animal experiment lasted for 60 days. During the experimental period, rabbits were fed the same standard iso-caloric/iso-nitrogenic diet. The basal diet compound and the computational analysis followed the nutritional requirements of rabbits from the National Research Council guidelines [21], as shown in Table 1.

#### 2.2.3. Physicochemical Analysis

Moisture, protein, fat, titratable acidity, total carbohydrates and pH values of ingredients and yogurt samples were analyzed by the methodology mentioned [22]. Meat composition (moisture, protein, ash and pH values) was determined in all groups as described by [22]. In addition, the total antioxidant activity of yogurt versions was assessed as described by Prieto et al. [23].

#### 2.2.4. Microbiological Examinations

Lactic acid bacteria (LAB) were counted in yogurt samples, according to Elliker et al. [24]. *Lb. delbrueckii* subsp. *bulgaricus* and *Str. thermophilus* in yogurt were enumerated as described by Ryan et al. [25]. Coliform bacteria, yeasts and molds were evaluated by Marshall [26] and Gadaga et al. [27], respectively.

#### 2.2.5. Growth Performance

Weaned rabbits in each replicate were weighed at 4, 8, and 12 weeks of age, using a digital scale, and the average daily weight gain [ADG (g/weaned rabbit)] was calculated. Five rabbits were taken randomly at the end of the experimental period from each treatment for all further analyses. The animals were slaughtered to evaluate the carcass characteristics and weight of internal organs. The carcass and inner organs were expressed as related to the final body weight. At the end of the experimental period, the animals were slaughtered to evaluate the carcass traits and weight of internal organs. The weights of each carcass, forelegs, saddle, hind legs, thoracic neck, kidney, lung, heart, liver, spleen, head and neck were recorded and expressed vis-à-vis the final body weight.

#### 2.2.6. Blood Biochemical Parameters 

The blood samples were obtained without anticoagulant, allowed to clot at 4 °C and centrifuged at 3000× for 10 min to retrieve the blood serum. The non-hemolyzed serum was collected and stored at −20 °C until the serum biochemical parameters were measured. Alanine aminotransferase (ALT) and aspartate aminotransferase (AST) were determined using the Morgenstern et al. [28] method. Serum total triglyceride, cholesterol, high-density lipoprotein cholesterol (HDL-c) and low-density lipoprotein cholesterol (LDL-c) were assessed using commercial kits developed by Pasteur laboratories (Egyptian American Co. for Services of Laboratory, Nasr City, Cairo, Egypt). Immunoglobulin M (Ig M), Immunoglobulin G (Ig G) and Immunoglobulin A (Ig A) were determined using the methodology reported by Wu et al. [29].

#### 2.2.7. Villus Morphology and Morphometry

Five segments of the mid-jejunum (3 cm) from each treatment were collected, fixed with formalin for 48 h, and paraffin embedded. Two sections (100 μm) from each sample were obtained, stained with hematoxylin for 1 min and counterstained with eosin for 10 s, to assess the maximum villus length (measured from above the crypt to the tip of the villus), villus width, goblet lining cells and submucosa/muscularis/serosa thickness. All target variables were measured by a camera (OLYMPUS, TH4-200; Tokyo, Japan) and computer-aided digital image pro plus (IPP) analysis software (Image-Pro plus 4.5, Media Cybernetics, Silver Spring, MD, USA).

#### 2.2.8. Statistical Analysis

All data were expressed as means with a standard error mean (SEM) and were subjected to analysis of variance (ANOVA) in a one-way analysis of variance, using SAS (Statistical Analysis System) software version 2004 [30]. The individual animal was considered the experimental unit and included one fixed effect of source type of trace minerals in the statistical model. Duncan multiple-range tests were used to define the differences among treatments. The applied static model is as follows:

yij = µ + Ti + eij(1)

where: yij is the observations, µ = general mean, Ti: effect of treatment, eij: random error.

## 3. Results

### 3.1. Physicochemical Properties of Ingredients Used in Produced Yogurt

Table 2 shows the physicochemical properties of ingredients used in the preparation of yogurt. Buffalo milk contained moisture, protein, fat, acidity, and total carbohydrate contents at levels of 87.86, 0.3.35, 1.2, 0.14 and 5.35%, respectively. Moisture content varied largely in the different ingredients, starting from 5.23 (Spirulina) to 87.86% (buffalo milk). The data show that Ca-CN powder contained the maximum (*p* < 0.05) protein content (81.75%). On the other hand, Spirulina contained the highest (*p* < 0.05) fat content (6.15%) followed by WPC (4.23%) then finally Ca-CN powders (0.85%).

### 3.2. Physicochemical Properties of Yogurt

Table 3 shows the chemical properties of yogurt samples from different preparations. Moisture content varied slightly from 86.00 to 86.87%. In all groups, the protein content ranged from 3.72 to 4.65%. Protein content was significantly higher (*p* < 0.05) in all yogurts fortified with different additives, evidently due to the high protein content in the different added ingredients (Ca-CN, WPC and Spirulina powders). Fat content in yogurt containing Spirulina powder was significantly higher (*p* < 0.05) than the other treatments due to the high-fat content in the additive. Titratable acidity and pH values were not significantly affected by additives (*p* > 0.05). Total carbohydrates varied from 5.69 to 6.76%.

### 3.3. Total Antioxidant Activity of Yogurt

**Figure 1:**
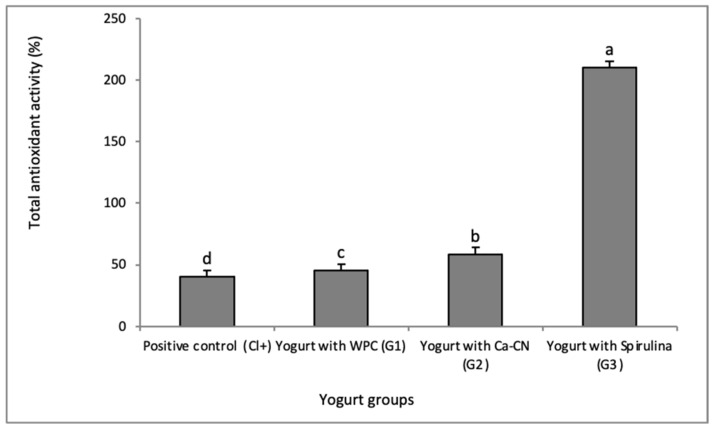
Total antioxidant activity (%) of yogurt supplemented with 1% of whey protein concentrate (G1), calcium caseinate (G2) and Spirulina powder (G3) as compared to a positive control (Cl+; yogurt without additives). Different letters (a, b, c, and d) within a column mark significantly different value (*p < 0.05*).

Total antioxidant (TA) values of yogurt samples are presented in Figure 1. Values expressing the total antioxidant activities ranged from 41.39 to 210.02%. The highest value (*p* < 0.05) occurred in G3 (yogurt fortified with Spirulina). Adding Spirulina to yogurt significantly (*p* < 0.05) and considerably increased the total antioxidants compared to the other treatments.

### 3.4. Microbiological Characteristics of Yogurt

Table 4 shows the differences in counts of lactic acid bacteria (LAB); *Str. thermophilus* and *Lb. delbrueckii* subsp. *bulgaricus*. In the different groups, LAB counts were of a limited range from 8.16 to 8.57 log_10_ CFU g^−1^. Followingly, the counts of *Str. thermophilus* varied from 7.91 to 8.12 log_10_ CFU g^−1^ in all samples and the counts of *Lb. delbrueckii* subsp. *bulgaricus* ranged from 8.01 to 8.38 log_10_ CFU g^−1^. 

### 3.5. Growth Performance

Growth performances of rabbits fed yogurts fortified with WPC, Ca-CN and Spirulina are shown in Table 5. Initial body weight of all groups after 4 weeks showed insignificant increases. However, the results show that the final body weight after 12 weeks of G1, G2 and G3 were significantly (*p* < 0.05) higher than both the negative and positive controls. The highest values of ADG 4–8 (g/d) were recorded in G1 and G2, i.e., receiving both WPC and Ca-Caseinate. The highest value of ADG 8–12 (g/d) was detected in G1 (receiving yogurt + 1% WPC). Additionally, Cl− (yogurt-free diet as a negative control) was higher than the Cl+ (non-supplemented yogurt as a positive control). On the other hand, the lowest level of ADG 8–12 (g/d) was observed in G2 (receiving yogurt + 1% Ca-caseinate) and Cl+ (as a positive control). However, the highest level of ADG 4–12 (g/d) at the end of the experiment was uniquely recorded in G1.

### 3.6. Carcass Traits

Table 6 shows the rabbits’ live body weight and carcass traits after 60 days of feeding on the tested yogurts. All animals were slaughtered at the end of the experiment to evaluate carcass characteristics and the carcass weights, liver, kidney, spleen, lung, heart and neck rates. Saddle, hind legs, thoracic neck, kidney, liver, spleen, head and neck percentages showed significant changes (*p* < 0.05) in all groups. The forelegs, lung and heart percentages of all groups were not significantly affected by additives.

### 3.7. Serum Biochemical Properties

The data in Table 7 show the serum biochemical properties of rabbits after 60 days of feeding on the tested yogurt compared to controls. There were no significant variations between all groups in the total cholesterol, AST and ALT values. However, contents of total protein, globulin and albumin between all groups showed significant (*p* < 0.05) differences. Total protein was significantly higher in the groups receiving fortified yogurts (G1, G2 and G3), particularly G1 (receiving WPC), which recorded the highest level. The density lipoprotein-cholesterol (HDL-c), creatinine, urea and IgM exhibited the highest (*p* < 0.05) levels in G1 and G3. Values of Immunoglobulin G (IgG), Immunoglobulin M (IgM) and Immunoglobulin A (IgA) were significantly (*p* > 0.05) higher in the groups receiving the fortified yogurts, particularly G1 and G3. On the other hand, the group receiving WPC-fortified yogurt (G1), indicated the lowest value of the low-density lipoprotein-cholesterol (LDL-c) (*p* < 0.05), as contrasted to the highest score noticed for the negative control (Cl−).

### 3.8. Villus Morphology and Morphometry

**Figure 2:**
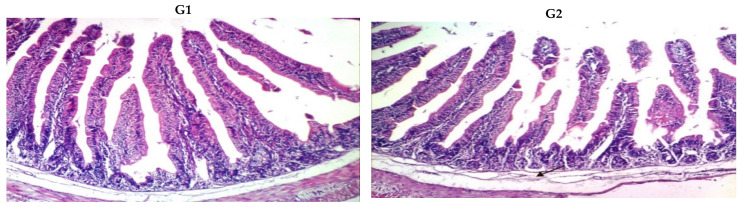

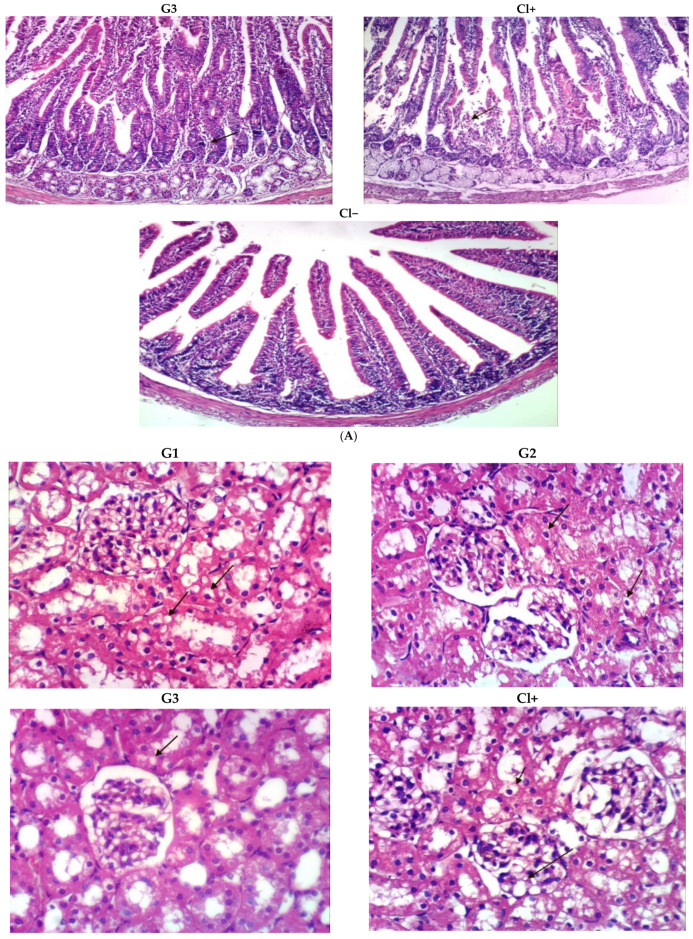

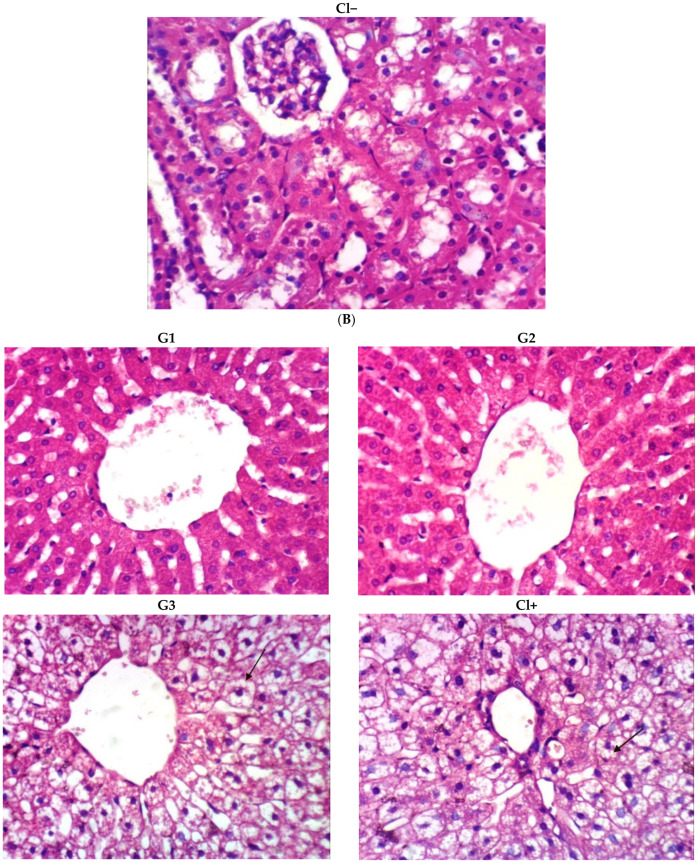

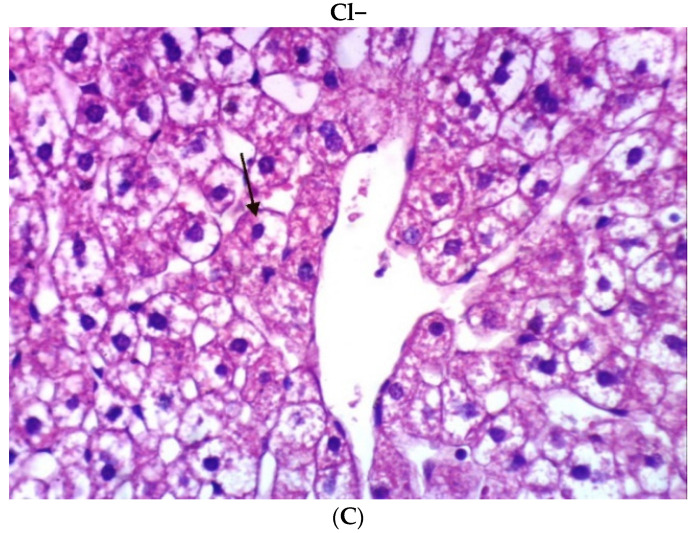
(**A**). Villus morphology and morphometry characteristics of the small intestine in the rabbits receiving yogurt fortified with 1% whey protein concentrate (G1), calcium caseinate (G2) and Spirulina powder (G3) as compared to that of non-supplemented yogurt (positive control: Cl+) or a yogurt-free diet (negative control (Cl−) (H & E X 100). The groups (G1–G3 and Cl+) received yogurt at a dose of 5 g/kg body weight/day. (**B**). Villus morphology and morphometry characteristics of the kidneys of rabbits receiving yogurt containing 1% whey protein concentrate (G1), calcium caseinate (G2), and Spirulina powder (G3) as compared to the non-supplemented yogurt (positive control: Cl+) or yogurt-free diet (negative control (Cl−) (H & E X 100). The groups (G1–G3 and Cl+) received yogurt at a dose of 5 g/kg body weight/day. (**C**). Villus morphology and morphometry examinations of the liver in rabbits receiving yogurt containing 1% whey protein concentrate (G1), calcium caseinate (G2) and Spirulina powder (G3) as compared the non-supplemented yogurt as a positive control (Cl+) or yogurt-free diet as a negative control (Cl−) (H & E X 100). The groups (G1–G3 and Cl+) received yogurt at a dose of 5 g/kg body weight/day.

Villus morphology and morphometry characteristics of all animal groups are shown in Figure 2. The small intestines of all groups show significant changes (Table 8 and Figure 2A). There were improvements (*p* < 0.05) in the small intestinal wall (intestinal layers from mucosa, submucosa, mucosa and serosa) of the groups receiving the fortified yogurts (G1, G2 and G3). The improvements (*p* < 0.05) in the small intestinal wall caused an increase of villus width (VW), villus length (VL), goblet lining cells (G Cell) and muscle thickness (MTh). However, the highest value (*p* < 0.05) was detected in Cl− for the number of villin section (NVIS) compared with the other groups. Figure 2B presents the kidney sections of all the animal groups. It can be seen that the histological characteristics of the kidney were not affected by additives in all groups, showing generally typical histological structures of the renal parenchyma. All groups showed vacuolar degeneration of epithelial lining renal tubules, endothelial lining glomerular tuft and congestion of renal blood vessels. Examined liver sections also indicate the absence of an effect of the additives in all groups, indicating globally regular histological structures of the hepatic lobule (Figure 2C). Rabbit liver showed a generally normal dilatation of hepatic sinusoids, congestion of hepatoportal blood vessels, binucleation of hepatocytes and pericholangiolar fibroblasts proliferation.

### 3.9. Meat Composition

Table 9 shows the meat quality of all groups after 60 days. Total solids, protein and pH values were significantly (*p* < 0.05) higher in rabbit meat receiving fortified yogurts (G1, G2 and G3) than the positive controls. This increase may be due to an increase in total solids and protein of the different additives. There were no significant differences in ash content for all groups. The yogurt enriched with WPC, Ca-CN and Spirulina generally improved meat quality.

## 4. Discussion

The data obtained on the physicochemical properties of ingredients used in preparing the fortified yogurts are similar to those reported by several authors [31,32]. The Spirulina was previously reported to contain moisture (3–7%), protein (55–60%), fat (6–8%), total carbohydrates (12–20%), ash (7–10%), chlorophy11 (1–1.5%) and vitamins. Some authors reported that whey protein isolates contained protein (93.2%), lactose (0.6%), fat (0.3%) and moisture (5.1%). Additionally, the sodium caseinate powder contained protein (93%), lactose (0.1%), fat (0.7%) and moisture (4.5%). The addition of whey protein isolates and sodium caseinate to yogurt was noticed to cause a significant increase in protein content but did not significantly affect the pH values. Generally, the data reflecting the physicochemical properties of the Spirulina-fortified yogurts are similar to those reported by Barkallah et al., particularly the higher level of protein content. These changes may be due to the high protein in Spirulina powder. Adding Spirulina powder to yogurt significantly changed the levels of moisture, ash, carbohydrate, fat and pH values [33]. Furthermore, Lee and Lucey [34] observed that adding milk protein powders to yogurt increased total solids. It was previously indicated that yogurt containing whey protein was associated with a more reduced pH than the control. The increase in total antioxidant activity in Spirulina-fortified yogurt can be ascribed to the antioxidant activity of Spirulina. Similar to those detected by Atallah et al. [35], reporting the most outstanding value of antioxidant activity corresponding to low-fat yogurt fortified with Spirulina. The increase in free radical scavenging may be due to the high carotenoids, chlorophylls and phycocyanin in Spirulina powder [36]. These findings agree with those reported by Barkallah et al. [37], who recorded the highest total protein in the yogurt enriched with Spirulina. Several animal and human studies have referred to the potential beneficial actions of Spirulina against several diseases such as cancer, anemia, leukemia, arterial hypertension and dyslipidemia [38,39]. Many of these beneficial effects may be due to the antioxidant activity of the additive or the enhanced synthesis of endothelial nitric oxide and the immuno-suppressive potential of the Spirulina [40,41]. The increase in the lactic acid bacteria count in some prepared Spirulina-fortified yogurts [38] are similar to those reported by Agustini et al. [33], who stated that adding *Spirulina platensis* to yogurt showed a positively enhanced growth rate of LAB. The coliform bacteria, yeasts and molds counts were not undetectable in all samples. This favorable action on LAB may be due to the high sanitation conditions and sufficient heat treatment of milk during yogurt production and the nutritive action of the additives.

The excellent growth performance of the rabbits’ groups receiving WPC- and Ca-CN- enriched yogurt (G1 and G2) may be due to the raised levels of protein content, implying bioactive peptides and essential amino acids. These findings are in agreement with those reported by Boirie et al. [42], and Frühbeck et al. [43], who found that WPC has high levels of sulfur amino acids (methionine and cysteine) and threonine, alanine and glycine. On the other hand, Ca-CN was explicitly high in some essential ones, e.g., histidine, isoleucine, leucine, lysine, phenylalanine and valine levels [43]. Milk proteins are the main source of a range of biologically active peptides, essential amino acids, and the appearance of their specific physiological characteristics may have nutritional significance, which is another aspect of their nutritional value [44,45,46,47,48,49,50,51,52]. These results are similar to those of Karkos et al. [53], who noticed significantly greater body weight when supplementing Spirulina into the poultry diet than the control. The highest value of ADG 8–12 (g/d) was detected in G1 (receiving yogurt + 1% WPC). Actually, animal growth is dependent upon the time and the availability of nutritional requirements. The positive control animals had access to additional food components from the added yogurt, including proteins and vitamins. This allowed this animal group (Cl+) to grow rapidly in the earlier stage (4–8 weeks), achieving higher ADG than the negative control. Since the growth of Cl+ achieved its maximum value at this stage, the next period (8–12 weeks) showed a relatively lower ADG value than the negative control. On the other hand, the negative control (without yogurt) with lower availability of nutritive elements, showed lower growth rate and ADG at the early stage (4–8 weeks). Then it tended to compensate in the late stage as a natural mechanism of growth, achieving higher ADG. However, the overall growth during the period (4–12 week) was higher in Cl+ than Cl−, being reflected on higher ADG values, due to the supplied nutritional elements. Thus, the noticed changes may have resulted from the ordinary fluctuations in the growth rate according to the prevailing nutritional conditions, but the overall ADG (4–12 weeks) favored the positive control. Further on, the lowest level of ADG 8–12 (g/d) was observed in G2 (receiving yogurt + 1% Ca-caseinate) and Cl+ (as a positive control). The increase in body weight can be related to an increase in essential fatty acids, amino acids, vitamins, minerals and other compounds of Spirulina. APRI doe rabbits and New Zealand White rabbit bucks fed Spirulina diet showed significantly increased growth performance parameters compared to the control group [54,55,56]. Spirulina diets are also known to contain *β*-carotene and other antioxidants and vitamins (A, C and E), which have regulative effects on animal physiology and fertility performance [57]. 

Whey protein and casein are considered to have the most nutritional value compared to other proteins. This increase in nutritional value can be related to an increase in essential amino acids, sulfur amino acids, and their good digestibility [58,59]. In addition to its nutritional value, whey protein concentrate was reported to exhibit antimicrobial activity against some human pathogens and spoilage bacteria in dairy products. Spirulina contains essential amino acids, vitamins and minerals. It is also a rich source of fatty acids and carotenoids [60]. However, the percentage of carcass, forelegs, lung and heart did not significantly (*p* > 0.05) change with the fortification with WPC, Ca-CN and Spirulina in comparison with the negative control. Additionally, Vonshak [61] found that the addition of whey powder to the broiler ration improved carcass weight, carcass percent, breast weight, drum stick weight and wings weight. Moreover, Vonshak [61] reported that whey protein concentrate lowered the cecal and intestinal pH. These decreases in the cecal and intestinal pH are considered related to the activity of the intestinal microorganisms of the rabbit intestine, including lactic acid bacteria, which can ferment lactose sugar, components of ingredients and produce volatile fatty acids in the intestinal lumen to decrease the pH of cecal and intestinal contents. 

The changes in the serum biochemical properties of the rabbits receiving the additives are in agreement with those reported by Khan [5], Blé-Castillo et al. [58] and Torres-Duran et al. [59], who observed the highest in serum protein values (albumin and globulin). This increase may be due to protein quality and quantity of WPC, Ca-CN and Spirulina. These results are similar to those reported by Kaur et al. [62], who indicated the highest decrease in total cholesterol, LDL-cholesterol, VDL-cholesterol and triglycerides contents in rats or mice fed Spirulina. This decrease may be due to the effect of Spirulina on lipoproteins metabolism and the increase of the lipoprotein enzyme activity levels [53]. Generally, tested serum biochemical parameters were significantly affected by supplementing the dietary Ca-CN, WPC and Spirulina. The effects of WPC, Ca-CN and Spirulina on the tested serum biochemical parameters may be due to the powerful antioxidant, amino acid, phycocyanin, tocopherols, beta carotene and vitamin C on the growth and health status of experimental animals [53,63,64]. The addition of WPC, Ca-CN and Spirulina improved the gut health (intestinal lumen, small intestinal and large intestinal) of rabbits and decreased the cecal content pH and intestinal pH. This decrease in pH can be related to the acid production activity of the intestinal organisms, including lactic acid bacteria, which occur through fermenting lactose to lactic acid, and volatile fatty acids in the intestinal lumen [65]. The results reflecting the improved quality of rabbit meat obtained after feeding on the enriched diets are consistent with previous studies [19,66,67,68], defining the Spirulina in the diet as the reason for increasing the ratio n-6/n-3 PUFA and CLA in the lipid content of muscle.

## 5. Conclusions

Supplementing yogurt with WPC 1%, Ca-CN 1% and *Spirulina platensis* 1% induced considerable impacts on the different quality attributes of rabbit performance and products. The addition of Spirulina significantly increased the total antioxidant activity compared to the other treatments. The final live body weight of G1, G2 and G3 was significantly (*p* < 0.05) higher than the other groups. However, percentages of saddle, hind legs, liver and neck were significantly (*p* < 0.05) affected by the additives. The LDL-cholesterol, total protein, globulin, albumin, creatinine and IgM levels were significantly (*p* < 0.05) decreased in G1 compared to the other groups. There were significant improvements (*p* < 0.05) in the small intestinal wall of the G1, G2 and G3 treatments. Villus morphology and morphometry characterizations of the kidney and liver were not affected by additives in all groups. Generally, the treatments G1, G2 and G3 significantly promoted the physicochemical, microbiological, growth performance, serum biochemical properties, and meat quality of rabbits.

## Figures and Tables

**Table 1:** Composition and calculated analyses of the basal diet (g/kg, as-fed basis) of weaned rabbits.

Ingredients	Content
Ingredients (g/kg)	
Alfalfa hay	350
Yellow corn	200
Soybean meal	96
Wheat bran	300
Corn stover	30
Di-calcium phosphate	12.5
L-Lysine HCl	2.1
DL-Methionine	2
Sodium chloride	5
Vitamin/mineral premix *	1.5
Total	1000.0
Calculated analysis (g/kg on dry matter basis)	
Digestible energy (MJ/kg)	11.6
Crude protein (g/kg)	179
Crude fiber (g/kg)	125
Crude fat (g/kg)	32.0
Ca (g/kg)	10.9
Available P (g/kg)	5.9
Methionine (g/kg)	4.2
Lysine (g/kg)	9.0

* Minerals and vitamins mixture supply/kg of diet: vit. A, 20,000 IU; vit. D3, 15,000 IU; vit. E, 8.33 g; vit. K, 0.33 g; vit. B1, 0.33 g; vit. B2, 1.0 g; vit. B6, 0.33 g; vit. B5, 8.33 g; vit. B12, 1.7 mg; pantothenic acid, 3.33 g; biotin, 33 mg; folic acid, 0.83 g; choline chloride, 200 g.

**Table 2:** Physicochemical properties of ingredients used in produced yogurt.

Ingredients	Moisture %	Protein %	Fat %	Acidity %	Total CHO * %	pH Value
Buffalo milk	87.86 ^a^	3.35 ^d^	1.20 ^c^	0.14 ^b^	5.35 ^c^	6.81 ^a^
Whey protein concentrate (WPC)	8.38 ^c^	68.91 ^b^	4.23 ^b^	1.65 ^a^	13.24 ^b^	3.74 ^b^
Calcium caseinate (Ca-CN)	9.12 ^b^	81.75 ^a^	0.85 ^c^	0.15 ^b^	1.19 ^d^	6.83 ^a^
Spirulina	5.23 ^d^	61.86 ^c^	6.15 ^a^	0.11 ^b^	19.25 ^a^	6.81 ^a^
*p*-value	0.006	0.003	0.007	0.005	0.004	0.004
SEM	0.022	0.382	0.073	0.007	0.417	0.017

* CHO: total carbohydrates. Means within the same column with different letters are significantly different (*p* < 0.05).

**Table 3:** Physicochemical properties of yogurt fortified with milk protein concentrates and Spirulina.

Yogurt Groups	Moisture %	Protein %	Fat %	Acidity %	Total CHO * %	pH Value
Yogurt without any supplements as a positive control (Cl+)	86.87 ^a^	3.72 ^b^	1.37 ^ab^	0.74 ^a^	5.69 ^d^	4.39 ^a^
Yogurt with 1% WPC (G1)	86.01 ^c^	4.53 ^a^	1.30 ^b^	0.72 ^a^	6.76 ^a^	4.34 ^a^
Yogurt with 1% Ca-CN (G2)	86.39 ^b^	4.65 ^a^	1.37 ^ab^	0.71 ^a^	6.25 ^c^	4.34 ^a^
Yogurt with 1% Spirulina (G3)	86.00 ^c^	4.31 ^a^	1.531 ^a^	0.70 ^a^	6.41 ^b^	4.40 ^a^
*p*-value	0.008	0.006	0.002	0.452	0.0001	0.453
SEM	0.152	0.023	0.012	0.022	0.040	0.107

CHO: total carbohydrates. ^a–d^ Means within the same column with different letters are significantly different (*p* < 0.05).

**Table 4:** Microbiological examinations (log_10_ CFU g^−1^) of yogurt fortified with milk protein concentrates and Spirulina powder.

Yogurt Groups	Lactic Acid Bacteria	*Str. thermophilus*	*Lb. delbrueckii* Subsp. *bulgaricus*
Yogurt without any supplements as a positive control (Cl+)	8.16 ^c^	7.91 ^c^	8.01 ^c^
Yogurt with 1% WPC (G1)	8.57 ^a^	8.03 ^ab^	8.38 ^a^
Yogurt with 1% Ca-CN (G2)	8.30 ^b^	8.08 ^a^	8.20 ^b^
Yogurt with 1% Spirulina (G3)	8.51 ^a^	8.12 ^a^	8.31 ^a^
*p*-value	0.008	0.009	0.006
SEM	0.117	0.104	0.092

^a–c^ Means within the same column with different letters are significantly different (*p* < 0.05).

**Table 5:** Growth performances of rabbits as affected by yogurt groups. All yogurt samples were administered to animals at a dose of 5 g/kg body weight/day.

Growth Parameters	Groups					SEM	*p*-Value
	Cl+	Cl−	G1	G2	G3		
BW 4 (g)	564.16	565.93	563.36	566.80	568.73	35.105	0.9997
BW 8 (g)	1041.63 ^b^	839.46 ^d^	1159.90 ^a^	1161.63 ^a^	956.36 ^c^	13.733	0.0001
BW 12 (g)	1655.63 ^d^	1545.06 ^e^	2050.60 ^a^	1765.13 ^b^	1692.40 ^c^	16.801	0.0001
ADG 4–8 (g/d)	17.05 ^b^	9.76 ^d^	21.29 ^a^	21.24 ^a^	13.84 ^c^	1.616	0.0001
ADG 8–12 (g/d)	21.93 ^c^	25.20 ^b^	31.81 ^a^	21.55 ^c^	26.28 ^b^	0.625	0.0001
ADG 4–12 (g/d)	19.49 ^c^	17.48 ^d^	26.55 ^a^	21.40 ^b^	20.06 ^cb^	0.762	0.0001

G1: Yogurt containing 1% whey protein concentrate; G2: with 1% calcium caseinate; and G3: with 1% Spirulina powder compared to that of Cl+: non-supplemented yogurt as a positive control or Cl−: yogurt-free diet as a negative control; BW 4: bodyweight 4 weeks; BW 8: bodyweight 8 weeks; BW 12: bodyweight 12 weeks; ADG 8–4: average daily gain from 4 to 8 weeks; ADG 8–12: average daily gain from 8 to 12 weeks; ADG 4–12: average daily gain from 4 to 12 weeks. Means within a row with different letters are significantly different (*p* < 0.05).

**Table 6:** Carcass traits relative to live bodyweight of rabbits as affected by yogurt supplementation with 1% of whey protein concentrate (WPC), Ca-caseinate (Ca-CN) and *Spirulina platensis*.

Parameters	Groups					SEM	*p*-Value
	Cl+	Cl−	G1	G2	G3		
Live body weight (g)	1655.63 ^d^	1545.06 ^e^	2050.53 ^a^	1765.13 ^b^	1692.40 ^c^	16.78	0.0001
Carcass rate (%)	53.89 ^a^	52.92 ^a^	55.58 ^a^	53.07 ^a^	55.15 ^a^	1.72	0.28
Fore legs rate (%)	14.15 ^a^	14.29 ^a^	14.12 ^a^	14.24 ^a^	14.46 ^a^	0.57	0.95
Saddle rate (%)	22.46 ^ab^	20.97 ^c^	22.70 ^ab^	22.18 ^bc^	23.70 ^a^	0.70	0.01
Hind legs rate (%)	36.94 ^bc^	35.56 ^c^	38.76 ^a^	38.16 ^ab^	37.29 ^b^	0.75	0.004
Thoracical neck rate (%)	10.05 ^ab^	9.49 ^b^	11.44 ^a^	9.81 ^b^	10.76 ^ab^	0.76	0.06
Kidney index (%)	1.48 ^ab^	1.55 ^a^	1.16 ^c^	1.40 ^b^	1.43 ^b^	0.05	0.0001
Lung index (%)	1.32 ^a^	1.31 ^a^	1.63 ^a^	1.31 ^a^	1.30 ^a^	0.17	0.17
Heart index (%)	0.62 ^a^	0.67 ^a^	0.70 ^a^	0.64 ^a^	0.76 ^a^	0.11	0.63
Liver index (%)	6.71 ^ab^	4.92 ^c^	7.01 ^ab^	6.52 ^b^	7.65 ^a^	0.54	0.01
Spleen index (%)	0.10 ^a^	0.07 ^b^	0.10 ^a^	0.07 ^b^	0.10 ^a^	0.01	0.005
Head index (%)	12.22 ^a^	13.06 ^a^	10.22 ^b^	13.09 ^a^	12.47 ^a^	0.85	0.01
Neak rate (%)	9.86 ^b^	9.26 ^b^	11.75 ^a^	10.91 ^a^	9.14 ^b^	0.57	0.001

Cl+: non-supplemented yogurt as a positive control; Cl−: yogurt-free diet as a negative control; G1: received yogurt with WPC 1%; G2: received yogurt with Ca-CN 1%; G3: received yogurt with Spirulina 1%. All yogurt groups were orally administered at a dose of 5 g/kg body weight/day. Means within the same column with different letters are significantly different (*p* < 0.05).

**Table 7:** Serum biochemical properties of rabbits as affected by yogurt containing 1% whey protein concentrate (G1), calcium caseinate (G2) and Spirulina powder (G3) compared to that of a non-supplemented yogurt as a positive control (Cl+) or a yogurt-free diet as a negative control (Cl−).

Blood Parameters	Groups					SEM	*p*-Value
	Cl+	Cl−	G1	G2	G3		
Total protein (g/dL)	7.90 ^b^	7.03 ^c^	9.36 ^a^	8.06 ^b^	8.13 ^b^	0.28	0.0001
Globulin (g/dL)	2.46 ^a^	1.53 ^b^	2.56 ^a^	1.70 ^ab^	2.20 ^ab^	0.44	0.06
Albumin (g/dL)	6.40 ^b^	5.50 ^b^	6.70 ^a^	6.03 ^ab^	5.96 ^b^	0.36	0.01
LDL-c (mg/dL)	89.33 ^b^	101.00 ^a^	81.66 ^b^	87.00 ^b^	55.66 ^c^	5.98	0.0001
HDL-c (mg/dL)	48.66 ^ab^	43.33 ^b^	66.66 ^a^	57.00 ^ab^	66.66 ^a^	11.77	0.12
Triglyceride (mg/dL)	105.33 ^a^	74.00 ^b^	114.00 ^a^	109.33 ^a^	82.00 ^b^	9.44	0.0001
Cholesterol (mg/dL)	157.33 ^a^	111.33 ^a^	165.00 ^a^	150.33 ^a^	120.66 ^a^	30.58	0.20
Creatinine (mg/dL)	1.26 ^b^	0.84 ^c^	1.60 ^a^	1.50 ^ab^	1.66 ^a^	0.12	0.0001
Urea (mg/dL)	38.33 ^b^	29.00 ^c^	48.33 ^a^	47.66 ^a^	49.66 ^a^	3.01	0.0001
AST (IU/L)	65.00 ^a^	60.66 ^a^	64.00 ^a^	62.66 ^a^	67.00 ^a^	7.17	0.85
ALT (IU/L)	59.00 ^a^	56.33 ^a^	59.66 ^a^	57.66 ^a^	62.66 ^a^	7.95	0.89
IgG (mg/mL)	993.66 ^b^	950.00 ^b^	1010.00 ^b^	980.00 ^b^	1082.33 ^a^	36.73	0.01
IgA (mg/mL)	226.66 ^b^	220.00 ^b^	249.66 ^ab^	245.33 ^ab^	276.66 ^a^	22.65	0.07
IgM (mg/mL)	133.33 ^b^	129.00 ^b^	162.00 ^a^	133.66 ^b^	156.33 ^a^	7.93	0.0001

The groups (G1–G3 and Cl+) received yogurt at a dose of 5 g/kg body weight/day or a yogurt-free diet as a negative control (Cl−). Means within the same row with different letters are significantly different (*p* < 0.05). LDL-c: low-density lipoprotein-cholesterol; HDL-c: High-density lipoprotein-cholesterol; AST: Aspartate aminotransferase; ALT: Alanine aminotransferase; IgG: Immunoglobulin G; IgA: Immunoglobulin A and IgM: Immunoglobulin M.

**Table 8:** Villus morphology and morphometry characterizations of rabbits as affected by yogurt containing 1% whey protein concentrate (G1), calcium caseinate (G2) and Spirulina powder (G3) compared to that of non-supplemented yogurt as a positive control (Cl+) or yogurt-free diet as a negative control (Cl−).

Parameters (µm)	Groups					SEM	*p*-Value
	Cl+	Cl−	G1	G2	G3		
No. of villin section (NVIC)	47.14 ^b^	90.57 ^a^	53.20 ^b^	54.00 ^b^	55.80 ^b^	12.62	0.0001
Villus width (VW)	102.75 ^bc^	89.25 ^c^	129.00 ^a^	116.25 ^ab^	130.50 ^a^	24.94	0.0005
Villus length (VL)	367.58 ^c^	397.50 ^c^	555.00 ^a^	474.58 ^b^	540.33 ^a^	69.29	0.0001
Musclaris thickness (MTh)	64.50 ^b^	70.50 ^b^	194.83 ^a^	73.50 ^b^	88.50 ^b^	40.77	0.0001
Goblet lining cells (G Cell)	19.91 ^a^	16.00 ^b^	16.91 ^ab^	20.25 ^a^	20.08 ^a^	3.83	0.01

The groups (G1–G3 and Cl+) received yogurt at a dose of 5 g/kg body weight/day. Means within a row with different letters are significantly different (*p* < 0.05). Means within a column with different letters are significantly different (*p* < 0.05).

**Table 9:** Meat composition of rabbits as affected by yogurt containing 1% whey protein concentrate (G1), calcium caseinate (G2) and Spirulina powder (G3) compared to that of non-supplemented yogurt as a positive control (Cl+) or a yogurt-free diet as a negative control (Cl−).

Treatments	Moisture %	Protein %	Ash %	pH Value
Cl+	74.567 ^b^	16.417 ^d^	1.690 ^a^	6.680 ^c^
Cl−	74.923 ^a^	16.160 ^e^	1.642 ^a^	6.733 ^b^
G1	72.807 ^e^	16.980 ^c^	1.693 ^a^	6.767 ^a^
G2	73.460 ^d^	18.260 ^a^	1.607 ^a^	6.617 ^e^
G3	73.837 ^c^	17.420 ^b^	1.640 ^a^	6.633 ^d^
*p*-value	0.0001	0.0001	0.614	0.0001
SEM	0.392	0.047	0.609	0.030

The groups (G1–G3 and Cl+) received yogurts at a dose of 5 g/kg body weight/day. Means within a column with different letters are significantly different (*p* < 0.05).

## Data Availability

Data available upon request.

## References

[1] Kücükcetin A. (2008). Effect of heat treatment and casein to whey protein ratio of skim milk on graininess and roughness of stirred yoghurt. Food Res. Int..

[2] Deeth H., Tamime A. (1981). Yogurt: Nutritive and therapeutic aspects. J. Food Prot..

[3] Trachoo N., Mistry V. (1998). Application of ultrafiltered sweet buttermilk and sweet buttermilk powder in the manufacture of nonfat and low fat yogurts1. J. Dairy Sci..

[4] Food and Agriculture Organization of the United Nations (2013). Dietary Protein Quality Evaluation in Human Nutrition: Report of an FAO Expert Consultation, Auckland, New Zealand, 31 March–2 April 2011.

[5] Khan Z., Bhadouria P., Bisen P. (2005). Nutritional and therapeutic potential of Spirulina. Curr. Pharm. Biotechnol..

[6] Osman A., Abd-Elaziz S., Salama A.S.A., Eita A.A. (2019). Health protective actions of phycocyanin obtained from an Egyptian isolate of Spirulina platensis on albino rats. EurAsian J. BioSci..

[7] Osman A., Salama A., Mahmoud K.E., Sitohy M. (2021). Alleviation of carbon tetrachloride-induced hepatocellular damage and oxidative stress in rats by Anabaena oryzae phycocyanin. J. Food Biochem..

[8] Holman B., Kashani A., Malau-Aduli A. (2012). Growth and body conformation responses of genetically divergent Australian sheep to Spirulina (*Arthrospira platensis*) supplementation. J. Exp. Agric. Int..

[9] Holman B., Malau-Aduli A. (2013). Spirulina as a livestock supplement and animal feed. J. Anim. Physiol. Anim. Nutr..

[10] Mirzaie S., Zirak-Khattab F., Hosseini S.A., Donyaei-Darian H. (2018). Effects of dietary Spirulina on antioxidant status, lipid profile, immune response and performance characteristics of broiler chickens reared under high ambient temperature. Asian Australas. J. Anim. Sci..

[11] Seyidoglu N., Galip N., Budak F., Uzabaci E. (2017). The effects of Spirulina platensis (*Arthrospira platensis*) and Saccharomyces cerevisiae on the distribution and cytokine production of CD4+ and CD8+ T-lymphocytes in rabbits. Austral J. Vet. Sci..

[12] Seyidoglu N., Inan S., Aydin C. (2017). A prominent superfood: Spirulina platensis. Superfood and Functional Food The Development of Superfoods and Their Roles as Medicine. Biology.

[13] Fouda F.S., Ismail R.F. (2017). Effect of Spirulina platensis on reproductive performance of rabbit bucks. Egypt. J. Nutr. Feed..

[14] Liisberg U., Myrmel L.S., Fjære E., Rønnevik A.K., Bjelland S., Fauske K.R., Holm J.B., Basse A.L., Hansen J.B., Liaset B. (2016). The protein source determines the potential of high protein diets to attenuate obesity development in C57BL/6J mice. Adipocyte.

[15] Zhu Y., Lin X., Zhao F., Shi X., Li H., Li Y., Zhu W., Xu X., Li C., Zhou G. (2015). Meat, dairy and plant proteins alter bacterial composition of rat gut bacteria. Sci. Rep..

[16] Smidt H., Lin X., Fan Zhao X.S., Li H., Li Y., Zhu W., Xu X., Li C., Zhou G. (2016). The gut microbiota and host health: A new clinical frontier. Gut.

[17] Gomes A.C., Hoffmann C., Mota J.F. (2018). The human gut microbiota: Metabolism and perspective in obesity. Gut Microbes.

[18] Zmora N., Suez J., Elinav E. (2019). You are what you eat: Diet, health and the gut microbiota. Nat. Rev. Gastroenterol. Hepatol..

[19] Zotte D.A., Szendrő Z. (2011). The role of rabbit meat as functional food. Meat Sci..

[20] Meineri G., Ingravalle F., Radice E., Aragno M. (2009). Peiretti Effects of high fat diets and Spirulina Platensis supplementation in New Zealand White rabbit. J. Anim. Vet. Adv..

[21] Smith S., Casady R., Donefer E. (1966). Nutrient requirements of rabbits. Nat. Acad. Sci. Nat. Res. Counc. Publ..

[22] Williams S. (1984). Official Methods of Analysis of the Association of Analytical Chemists.

[23] Prieto P., Pineda M., Aguilar M. (1999). Spectrophotometric quantitation of antioxidant capacity through the formation of a phosphomolybdenum complex: Specific application to the determination of vitamin E. Anal. Biochem..

[24] Elliker P., Anderson A., Hannesson G. (1956). An agar culture medium for lactic acid streptococci and lactobacilli. J. Dairy Sci..

[25] Ryan M.P., Rea M.C., Hill C., Ross R.P. (1996). An application in cheddar cheese manufacture for a strain of Lactococcus lactis producing a novel broad-spectrum bacteriocin, lacticin 3147. Appl. Environ. Microbiol..

[26] Marshall R.T. (1992). Standard Methods for the Examination of Dairy Products.

[27] Gadaga T., Mutukumira A., Narvhus J. (2000). Enumeration and identification of yeasts isolated from Zimbabwean traditional fermented milk. Int. Dairy J..

[28] Morgenstern S., Oklander M., Auerbach J., Kaufman J., Klein B. (1966). Automated determination of serum glutamic oxaloacetic transaminase. Clin. Chem..

[29] Wu Y., Zhu C., Zhanga Y., Li Y., Sun J. (2019). Immunomodulatory and antioxidant effects of pomegranate peel polysaccharides on immunosuppressed mice. Int. J. Biol. Macromol..

[30] SAS Institute (1996). SAS/STAT Software: Changes and Enhancements for Release 6.12.

[31] Guzmán-González M., Morais F., Ramos M., Amigo L. (1999). Influence of skimmed milk concentrate replacement by dry dairy products in a low fat set-type yoghurt model system. I: Use of whey protein concentrates, milk protein concentrates and skimmed milk powder. J. Sci. Food Agric..

[32] Remeuf F., Mohammed S., Sodini I., Tissierb J.P. (2003). Preliminary observations on the effects of milk fortification and heating on microstructure and physical properties of stirred yogurt. Int. Dairy J..

[33] Agustini T., Soetrisnanto D., Ma’ruf W. (2017). Study on chemical, physical, microbiological and sensory of yoghurt enriched by Spirulina platensis. Int. Food Res. J..

[34] Lee W.-J., Lucey J. (2010). Formation and physical properties of yogurt. Asian Australas. J. Anim. Sci..

[35] Atallah A.A., Morsy O.M., Gemiel D.G. (2020). Characterization of functional low-fat yogurt enriched with whey protein concentrate, Ca-caseinate and spirulina. Int. J. Food Prop..

[36] Ismaiel M.M.S., El-Ayouty Y.M., Piercey-Normore M. (2016). Role of pH on antioxidants production by Spirulina (Arthrospira) platensis. Braz. J. Microbiol..

[37] Barkallah M., Dammak M., Louati I., Hentati F., Hadrich B., Mechichi T., Ayadi M.A., Fendri I., Attia H., Abdelkafi S. (2017). Effect of Spirulina platensis fortification on physicochemical, textural, antioxidant and sensory properties of yogurt during fermentation and storage. LWT.

[38] Juárez-Oropeza M.A., Mascher D., Torres-Durán P.V., Farias J.M., Paredes-Carbajal M.C. (2009). Effects of dietary Spirulina on vascular reactivity. J. Med. Food.

[39] Grawish M.E., Zaher A.R., Gaafar A.I., Nasif W.A. (2010). Long-term effect of Spirulina platensis extract on DMBA-induced hamster buccal pouch carcinogenesis (immunohistochemical study). Med. Oncol..

[40] Dartsch P.C. (2008). Antioxidant potential of selected Spirulina platensis preparations. Phytother. Res. Int. J. Devoted Pharmacol. Toxicol. Eval. Nat. Prod. Deriv..

[41] Park H.J., Lee Y.J., Ryu H.K., Kim M.H., Chung H.W., Kim W.Y. (2008). A randomized double-blind, placebo-controlled study to establish the effects of spirulina in elderly Koreans. Ann. Nutr. Metab..

[42] Boirie Y., Dangin M., Gachon P., Vasson M., Maubois J., Beaufrère B. (1997). Slow and fast dietary proteins differently modulate postprandial protein accretion. Proc. Natl. Acad. Sci. USA.

[43] Frühbeck G., Jebb S., Prentice A. (1998). Leptin: Physiology and pathophysiology. Clin. Physiol..

[44] Sindayikengera S., Xia W.-s. (2006). Nutritional evaluation of caseins and whey proteins and their hydrolysates from Protamex. J. Zhejiang Univ. Sci. B.

[45] Chobert J.M., Sitohy M., Whitaker J.R. (1987). Specific limited hydrolysis and phosphorylation of food proteins for improvement of functional and nutritional properties. J. Am. Oil Chem. Soc..

[46] Abdel-Hamid M., Goda H.A., de Gobba C., Jenssen H., Osman A. (2016). Antibacterial activity of papain hydrolysed camel whey and its fractions. Int. Dairy J..

[47] Abdel-Hamid M., Osman A., El-Hadary A., Romeih E., Sitohy M., Li L. (2020). Hepatoprotective action of papain-hydrolyzed buffalo milk protein on carbon tetrachloride oxidative stressed albino rats. J. Dairy Sci..

[48] Abdel-Hamid M., Romeih E., Saporito P., Osman A., Mateiu R.V., Mojsoska B., Jenssen H. (2020). Camel milk whey hydrolysate inhibits growth and biofilm formation of Pseudomonas aeruginosa PAO1 and methicillin-resistant Staphylococcus aureus. Food Control..

[49] Abdel-Hamid M., Otte J., de Gobba C., Osman A., Hamada E. (2017). Angiotensin I-converting enzyme inhibitory activity and antioxidant capacity of bioactive peptides derived from enzymatic hydrolysis of buffalo milk proteins. Int. Dairy J..

[50] Kishawy A.T., Amer S.A., Osman A., Elsayed S.A.M., El-Hack M.E.A., Swelum A.A., Ba-Awadh H., Saadeldin M.I. (2018). Impacts of Supplementing Growing Rabbit Diets with Whey Powder and Citric Acid on Growth Performance, Nutrient Digestibility, Meat and Bone Analysis, and Gut Health. AMB Express.

[51] Ashour E.A., El-Hack M.E.A., Alagawany M., Swelum A.A., Osman A.O., Saadeldin I.M., Abdel-Hamid M., Hussein E.O.S. (2019). Use of whey protein concentrates in broiler diets. J. Appl. Poult. Res..

[52] Osman A., El-Hadary A., Korish A.A., AlNafea H.M., Alhakbany M.A., Awad A.A., Abdel-Hamid M. (2021). Angiotensin-I Converting Enzyme Inhibition and Antioxidant Activity of Papain-Hydrolyzed Camel Whey Protein and Its Hepato-Renal Protective Effects in Thioacetamide-Induced Toxicity. Foods.

[53] Karkos P., Leong S.C., Karkos C.D., Sivaji N., Assimakopoulos D.A. (2011). Spirulina in clinical practice: Evidence-based human applications. Evid.-Based Complement. Altern. Med..

[54] Dalle Zotte A., Sartori A., Bohatir P., Rémignon H., Riccia R. (2013). Effect of dietary supplementation of Spirulina (*Arthrospira platensis*) and Thyme (*Thymus vulgaris*) on growth performance, apparent digestibility and health status of companion dwarf rabbits. Livest. Sci..

[55] Gerencser A.A., Chinopoulos C., Birket M.J., Jastroch M., Vitelli C., Nicholls D.G., Brand M.D. (2012). Quantitative measurement of mitochondrial membrane potential in cultured cells: Calcium-induced de-and hyperpolarization of neuronal mitochondria. J. Physiol..

[56] El-Desoky G.E., Bashandy S.A., Alhazza I.M., Al-Othman Z.A., Aboul-Soud M.A.M., Yusuf K. (2013). Improvement of mercuric chloride-induced testis injuries and sperm quality deteriorations by Spirulina platensis in rats. PLoS ONE.

[57] RaMadaN M.F., Selim aSkeR M.M. (2008). Functional bioactive compounds and biological activities. Czech J. Food Sci..

[58] Blé-Castillo J., Rodríguez-Hernández A., Miranda-Zamora R., Juárez-Oropeza M.A., Díaz-Zagoya J.C. (2002). Arthrospira maxima prevents the acute fatty liver induced by the administration of simvastatin, ethanol and a hypercholesterolemic diet to mice. Life Sci..

[59] Torres-Duran P.V., Ferreira-Hermosillo A., Juarez-Oropeza M.A. (2007). Antihyperlipemic and antihypertensive effects of Spirulina maxima in an open sample of Mexican population: A preliminary report. Lipids Health Dis..

[60] Howe P., Meyer B., Record S., Baghurst K. (2006). Dietary intake of long-chain ω-3 polyunsaturated fatty acids: Contribution of meat sources. Nutrition.

[61] Vonshak A. (1997). Spirulina Platensis Arthrospira: Physiology, Cell-Biology and Biotechnology.

[62] Kaur K., Sachdeva R., Grover K. (2008). Effect of supplementation of Spirulina on blood glucose and lipid profile of the non-insulin dependent diabetic male subjects. J. Dairy. Foods Home Sci..

[63] Abdel-Daim M.M., Abuzead S.M., Halawa S.M. (2013). Protective role of Spirulina platensis against acute deltamethrin-induced toxicity in rats. PLoS ONE.

[64] Seyidoglu N., Gurbanli R., Köşeli E., Cengiz F. (2019). The effects of Spirulina (Arthrospira) platensis on morphological and hematological parameters evoked by social stress in male rats. J. Istanb. Vet. Sci..

[65] Ha E., Zemel M.B. (2003). Functional properties of whey, whey components, and essential amino acids: Mechanisms underlying health benefits for active people. J. Nutr. Biochem..

[66] Peiretti P., Meineri G. (2008). Effects of diets with increasing levels of Spirulina platensis on the performance and apparent digestibility in growing rabbits. Livest. Sci..

[67] Imbabi T., Hassan A., Ahmed-Farid O., El-Garhy O., Sabeq I., Moustafa M., Mohammadein A., Hassan N., Osman A., Sitohyg M. (2021). Supplementing rabbit diets with butylated hydroxyanisole affects oxidative stress, growth performance, and meat quality. Animal.

[68] Osman A., Imbabi T.A., El-Hadary A., Sabeq I.I., Edris S.N., Merwad A., Azab E., Gobouri A.A., Mohammadein A., Sitohy M. (2021). Health Aspects, Growth Performance, and Meat Quality of Rabbits Receiving Diets Supplemented with Lettuce Fertilized with Whey Protein Hydrolysate Substituting Nitrate. Biomolecules.

